# AxonSeg: Open Source Software for Axon and Myelin Segmentation and Morphometric Analysis

**DOI:** 10.3389/fninf.2016.00037

**Published:** 2016-08-19

**Authors:** Aldo Zaimi, Tanguy Duval, Alicja Gasecka, Daniel Côté, Nikola Stikov, Julien Cohen-Adad

**Affiliations:** ^1^Institute of Biomedical Engineering, Polytechnique MontrealMontreal, QC, Canada; ^2^Institut Universitaire en Santé Mentale de QuébecQuebec, QC, Canada; ^3^Centre d’Optique, Photonique et Laser, Université LavalQuebec, QC, Canada; ^4^Montreal Heart InstituteMontreal, QC, Canada; ^5^Functional Neuroimaging Unit, CRIUGM, Université de MontréalMontreal, QC, Canada

**Keywords:** axon, myelin, segmentation, discriminant analysis, histology, microscopy, graphical user interface, g-ratio

## Abstract

Segmenting axon and myelin from microscopic images is relevant for studying the peripheral and central nervous system and for validating new MRI techniques that aim at quantifying tissue microstructure. While several software packages have been proposed, their interface is sometimes limited and/or they are designed to work with a specific modality (e.g., scanning electron microscopy (SEM) only). Here we introduce AxonSeg, which allows to perform automatic axon and myelin segmentation on histology images, and to extract relevant morphometric information, such as axon diameter distribution, axon density and the myelin g-ratio. AxonSeg includes a simple and intuitive MATLAB-based graphical user interface (GUI) and can easily be adapted to a variety of imaging modalities. The main steps of AxonSeg consist of: (i) image pre-processing; (ii) pre-segmentation of axons over a cropped image and discriminant analysis (DA) to select the best parameters based on axon shape and intensity information; (iii) automatic axon and myelin segmentation over the full image; and (iv) atlas-based statistics to extract morphometric information. Segmentation results from standard optical microscopy (OM), SEM and coherent anti-Stokes Raman scattering (CARS) microscopy are presented, along with validation against manual segmentations. Being fully-automatic after a quick manual intervention on a cropped image, we believe AxonSeg will be useful to researchers interested in large throughput histology. AxonSeg is open source and freely available at: https://github.com/neuropoly/axonseg.

## Introduction

The central nervous system, which consists of the brain and the spinal cord, relies on the transmission of electrical signals via white matter axons. The myelin sheath, wrapped around the axons, has a key role in the transmission process over long distances (Zoupi et al., 2011; Seidl, 2014). In case of degenerative diseases such as multiple sclerosis, myelin tends to degenerate by undergoing significant morphological changes, affecting signal propagation (Lassmann, 2014; Alizadeh et al., 2015; Papastefanaki and Matsas, 2015). A large body of research focuses on the understanding of the intrinsic patterns related to demyelination in animal models (e.g., Experimental Autoimmune Encephalomyelitis, shivered, Wallerian degeneration; Baker and Amor, 2014; Ben-Nun et al., 2014; Papastefanaki and Matsas, 2015). Therefore, it is of particular interest to image white matter microstructure with high enough resolution to identify axon and myelin morphology. Histology has provided valuable information, but popular imaging techniques such as transmission electron microscopy (TEM) can only image small regions (typically ~100 × 100 μm^2^). New imaging techniques with a sliding acquisition window and stitching capabilities have emerged that can provide a full picture of a sample under investigation, e.g., a 1 × 1 cm^2^ cross-section of a spinal cord. However, axon and myelin segmentation of these large datasets is extremely time-consuming and difficult, as a dataset covering several cm^2^ contains millions of axons. Moreover, manual segmentation is subject to user bias and is therefore not reproducible within and across sites.

The first software tools capable of accomplishing segmentation of nerve fibers have mostly focused on simple segmentation algorithms for microscopic images stained with toluidine blue (Cuisenaire et al., 1999; Romero et al., 2000). Some research groups have opted for manual segmentation (Berthold et al., 1983; Dula et al., 2010; Liewald et al., 2014). Liewald et al. (2014) performed manual segmentation on TEM samples in order to study the distribution of axons diameters in the cortical white matter. Begin et al. (2014) introduced an algorithm capable of segmenting both axon and myelin in large-scale images from coherent anti-Stokes Raman scattering (CARS) microscopy. Their software can be used to extract morphological data from the input images and is fully automated. Although their software could be used on different contrasts, all the parameters are set to work on CARS images, and no graphical user interface (GUI) is included to adapt them to other contrasts. More et al. (2011) introduced a simple semi-automated algorithm designed to work on scanning electron microscopy (SEM) images. While a GUI is included, its function is limited to segmentation parameters only, which are hard to provide without prior knowledge of the tissue sample to segment. Moreover, the axon segmentation needs to be manually corrected before launching the myelin sheath segmentation and there is no integrated framework for extracting relevant morphometric information afterwards. Other segmentation tools focus on TEM images from optic nerves (Zhao et al., 2010) or cross sectional images of rat nerve fibers from the sciatic nerve in optical microscopy (OM; Wang et al., 2012). However, both work only on specific imaging modalities.

Most of the published work describes the segmentation algorithms without giving open access to the related scripts, or without providing an intuitive interface for other researchers to use. In summary, there is no single software for axon/myelin segmentation that is adapted to all imaging contrasts, is available for free, handles large-scale histology data and has a GUI. Having such software would facilitate the processing of large microscopy images and standardize processing across research groups.

In this article, we introduce AxonSeg, which is designed to perform axon and myelin segmentation on large-scale histology images, features an intuitive GUI, works with various contrasts and is open source. This article is organized as follows: (i) the “Methods” Section lists the main steps of AxonSeg, details the segmentation strategies and the discrimination model, then details the validation part; (ii) the “Results” Section presents validation results, proof-of-concept axon and myelin segmentation obtained from three different contrasts (CARS, OM and SEM) and shows statistics results on relevant morphological metrics from the input images; (iii) the “Discussion” Section addresses pros/cons of AxonSeg and discusses further possible developments; and (iv) an example use case describes the typical workflow in order to segment an OM sample.

## Materials and Methods

### Algorithm

AxonSeg aims at performing both axon and myelin segmentation on various imaging contrasts, including a robust axon candidate discrimination step that aims at optimizing sensitivity and precision.

#### Axon Segmentation

The axon segmentation strategy is based on Begin et al. (2014) and uses the extended minima method (also known as gradient-based region growing method) to output binary segmentations of the intracellular part of the axon (i.e., axon without myelin). The extended minima algorithm is defined as the regional minima of the H-minima transform. The H-minima transform eliminates all minima whose intensity is less than input threshold *h*. The regional minima, i.e., connected components of pixels with the same intensity value, and whose outer boundary pixels have higher values, are then computed.

The binary axon segmentation image is then post-processed by common morphological operations: remove isolated individual pixels, fill isolated interior pixels, perform morphological closing, remove H-connected pixels, perform morphological opening and finally, remove all partial axon candidates that touch the outside border.

#### Axon Discrimination

During the axon segmentation step, false positives (FPs) are inevitably introduced in the resulting output image. Thus, a discrimination step is needed to keep most of the true axons while trying to reject the false ones. Our discrimination strategy aims at combining shape (morphology) and intensity features in order to build a discriminant analysis (DA) classifier that distinguishes true/false axons.

The DA model is initiated by using a training dataset as input. This training dataset is generated by the user using the procedure described in section “discriminant analysis” and is made of two groups: one group contains true axons and the other group contains false axons. Shape features are determined for every labeled object of the training groups:

– Circularity: describes the roundness of the object, defined as 4π × Area/Perimeter^2^;
– Solidity: describes the compactness of the object, computed as Area/ConvexArea, where the convex area is the area of the polygon containing the object;
– Ellipticity: describes the flattening of the object, defined as the ratio between the minor and major axes;
– Equivalent diameter: diameter of the object, computed as the diameter of a circle with the same area as the object.

In a similar way, intensity features are computed for every labeled object of the training groups:

– Object intensity mean and standard deviation;
– Neighborhood intensity mean and standard deviation: the neighborhood is defined by performing a small morphological dilation of the object (disk-shaped structuring element with a radius of 2 pixels);
– Contrast: intensity difference between object and neighborhood intensity means.

True axons are usually described as round, convex, low intensity shapes, enclosed by a higher intensity myelin sheath annulus. The output of this process is a linear or quadratic classifier in the n-parameters space that can predict true/false axons from the untrained dataset.

#### Myelin Segmentation

The myelin segmentation strategy is based on the algorithm developed by Begin et al. (2014) which relies on radial screening of the axon neighborhood and the minimal-path algorithm (Vincent, 1998). First, after labeling the axons, the gradients of the radial profiles of each axon are computed by using a Sobel filter. The minimal-path algorithm is applied on radial profile gradients in order to detect the outer border of the myelin. In our implementation, we apply a subsequent maximal path to detect the inner border of the myelin sheath in order to refine the axon segmentation. Also, additional constraints have been added in order to improve the robustness of the myelin segmentation. This was done using a double snake algorithm (active contours) adapted from MATLAB Central File Exchange^1^, designed to detect both the outer and the inner boundary of the myelin sheath, and constrained to have a homogenous myelin thickness across the axon circumference (More et al., 2011) and a g-ratio (defined as the ratio of the inner to the outer diameter of the myelin sheath) within the range 0.4 and 1.

Next, a cleaning step is done by verifying the presence of conflicts between adjacent myelin areas. If more than 50% of the myelin sheath area from one axon is overlapping with the myelin sheath area from another adjacent axon, then the former axon is rejected from the analysis. The final axon segmentations are obtained from the corresponding myelin segmentations after computing a morphological filling.

### AxonSeg Software

**Figure 1:**
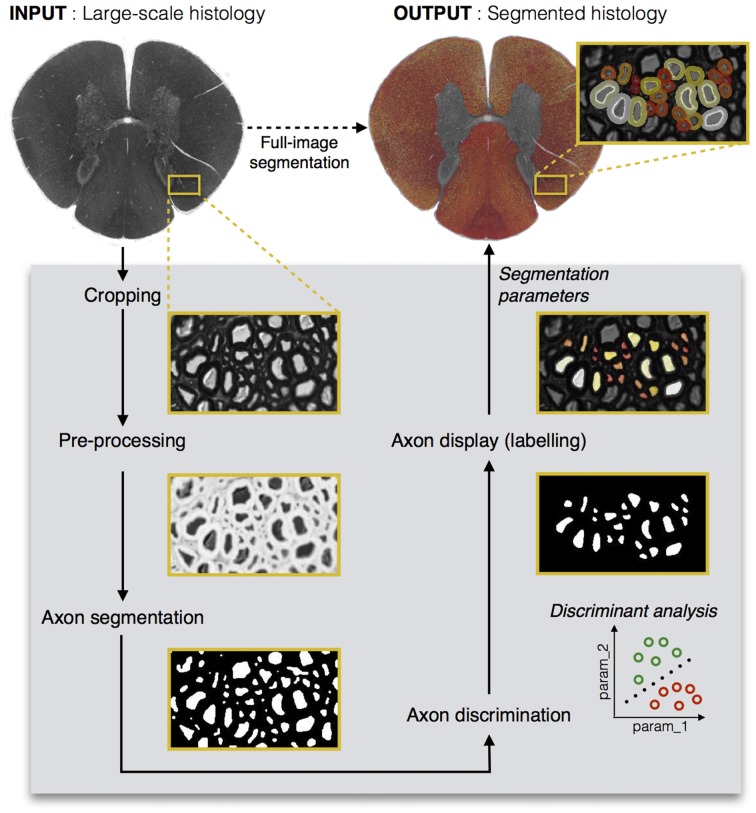
**Diagram illustrating the main steps of AxonSeg.** Pre-processing, axon segmentation and discrimination are first performed on a cropped image. Then, the segmentation parameters applied on the cropped image are saved and can be used to launch the automatic full-scale segmentation of the axons and the corresponding myelin sheaths. Note that axons within a range of 2 μm from the edge of the image are discarded to prevent the segmentation of incomplete axons.

Figure 1 shows the main steps in AxonSeg: cropping of a small region, image pre-processing, axon segmentation and DA, and axon and myelin segmentation of the full image. The main GUI tool of AxonSeg can be accessed by launching the *SegmentationGUI* function. Note that if the image is already small (e.g., less than 2000 × 2000 pixels), it is possible to bypass the cropping step and the DA step and just run the segmentation over the full image. Each step has a previewing capability and is composed of a “Go to next step” button that uses the selected parameters/options to generate and display the output. A “Reset Step” button allows the user to go back to the previous step.

#### Pre-Processing of Cropped Image

At the beginning of the process, the user has access to the following pre-processing tools: (i) a color inversion (complement) module to ensure that axons are darker than the myelin, as required by the segmentation strategy; (ii) a smoothing module (averaging filter, size 3 × 3); and (iii) contrast enhancement via histogram equalization and deconvolution. The user is asked to enter the pixel size, so that all morphological measurements regarding axon or myelin are given in distance units. If a scaling bar is present on the image (sometimes integrated from microscope outputs), the user can define the size of the scaling bar and select the two extreme points of this bar to get the pixel size.

#### Axon Segmentation From Cropped Image

Axon segmentation is performed using the extended minima algorithm, as explained in Section “Axon Segmentation”. The user can adjust segmentation results in real time by tuning the parameters of the extended minima (threshold values) with a slider (see “Example Use Case” Section for a typical use case).

#### Discriminant Analysis

A first global discrimination step, conducted by the user, aims at cleaning up the axon candidates field by eliminating a significant number of FPs. The “Minimal size” slider is used to remove FPs associated with small debris or artifacts (e.g., holes naturally present in the tissue), while the “Solidity” and “Ellipticity” sliders can help eliminate FPs coming from inter-nerve-fiber regions (extended intercellular spaces) of the image. The “Go to next step” button combines the result of each slider. On the next step, the user can manually remove the remaining FPs by clicking on the image. The selection of the features for the prior axon discrimination was made in accordance with the parameters analysis performed on control datasets. Similar feature analysis was performed by other research groups in order to find the best parameters that can separate the true positives (TPs) and FPs (Romero et al., 2000; Zhao et al., 2010; More et al., 2011; Wang et al., 2012; Begin et al., 2014), but none of them were able to find a perfect combination due to the large variability among axon shapes and intensities.

**Figure 2:**
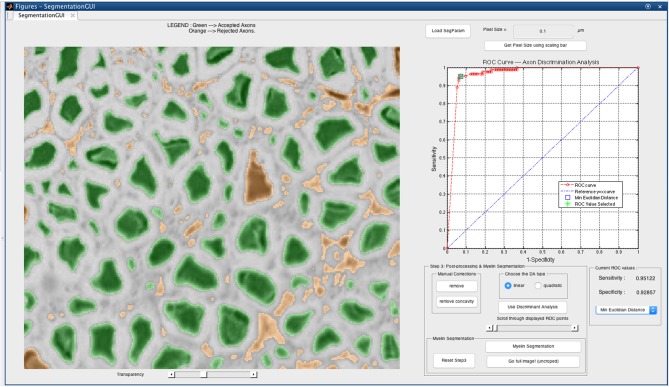
**Discriminant analysis (DA) step in the AxonSeg graphical user interface (GUI).** The user can scroll through available sensitivity and specificity combinations and display the results. For each sensitivity/specificity value, accepted axons are displayed in green while rejected axons are displayed in orange. The user can also decide to select a receiver operating characteristic (ROC) metric (e.g., minimal Euclidian distance, maximal sensitivity, maximal specificity). When satisfied with the DA classifier, the user can launch the myelin segmentation.

The DA tool is then launched on the corrected cropped image: the remaining axons are considered as TPs while those eliminated earlier are considered as FPs. The user can select either the linear DA or the quadratic one: the classification results will be displayed on the GUI, and the user can scroll through the “discriminant analysis” slider in order to select the sensitivity and specificity combination adapted to their needs. Opting for a higher sensitivity is a way to keep more axons, although it usually leads to lower specificity, thus accepting more FPs in the axon segmentation output. In our application, we aim at obtaining a classifier with maximal sensitivity and maximal specificity, i.e., the closest possible to the upper left corner of the receiver operating characteristic (ROC) curve. The minimal Euclidean distance from the upper left corner can thus be computed, and is available in the GUI along with other options (e.g., maximal sensitivity, maximal specificity). Figure 2 shows a screen capture of the GUI during the DA step.

Note that we do not let the user add missing axons in the GUI at this step for two main reasons. First, manually adding missed axons can add bias in shapes and thus strongly affect the DA. Secondly, AxonSeg comes with a smaller GUI tool called *ManualCorrectionGUI*, which can be used in order to correct a segmentation result by adding, removing or modifying axons after the full-image segmentation.

#### Myelin Segmentation on Cropped Image

The myelin segmentation is performed on the accepted axons (after manual correction or as determined by the selected DA result), as explained in Section “Myelin Segmentation”. The results are displayed on the GUI. The user can go back to previous steps and can adjust the parameters iteratively until satisfied with the final result.

#### Outputs

**Figure 3:**
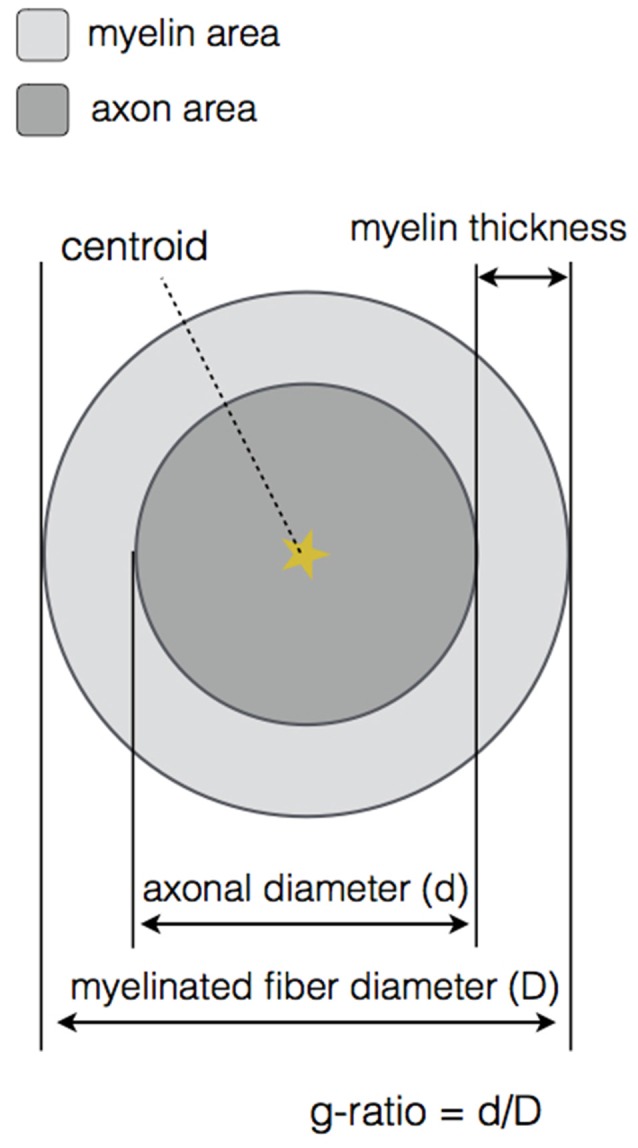
**Main morphological properties computed by AxonSeg.** For each myelinated fiber, the centroid, the axon and myelin areas, the axonal diameter, the myelinated fiber diameter, the myelin thickness and the myelin g-ratio are calculated.

At the end of the DA and myelin segmentation on the cropped image, an output folder is created. A very important feature of our software is the *axonlist* structure, which stores for each axon object the following fields: the data (all the pixels belonging to the axon object, in *x* and *y* coordinates), the axon and myelin areas (in both pixels and μm^2^), the centroid (in *x* and *y* coordinates), the axon ID (from labeling), the myelin g-ratio, the axon equivalent diameter (in μm), the myelinated fiber equivalent diameter (in μm) and the myelin thickness (in μm). These metrics are also stored in a comma-separated values (CSV) file, also in the output folder. Figure 3 illustrates the main morphological metrics computed by AxonSeg.

The default segmentation images saved in the output folder are labeled in axon equivalent diameter color code: a first one displaying the axon segmentation and a second one displaying the myelin segmentation. Other segmentation displays can be produced by using the *as_display_label* function included in AxonSeg. Two display types are available (either axon or myelin), color mapped in axon equivalent diameter, g-ratio, myelin thickness or axon ID. Additional images are also saved in the output folder: pre-processed input cropped image, binary image of the initial axon segmentation candidates and binary image of the final axon segmentation result after discrimination.

All parameters that have been adjusted using the GUI are saved into the MATLAB structure *SegParameters.mat* which is subsequently used for the full image segmentation. This structure can also be loaded in the GUI at any time if needed, in particular if the user wants to segment another image with similar contrast. Relevant parameters include pre-processing options, axon segmentation thresholds, prior axon discrimination values and DA classifiers.

#### Full Image Segmentation

If the user worked on a cropped region of the initial image, the full image segmentation can be launched by applying the same parameters as those used throughout the segmentation and processing of the cropped region. To avoid RAM saturation, the full image is processed by smaller blocks, with 20% overlap, and then stitched together. Note that this feature allows the segmentation of much larger images than the initial implementation by Begin et al. (2014) (images up to 21,000 × 12,000 pixels were processed successfully). This segmentation per block also allows parallelization across CPUs by using the Parallel Computing Toolbox in MATLAB (*parfor* and *parpool*), if supported by the computer.

This full segmentation can be run without using the GUI, by calling the *as_Segmentation_full_image* function, which requires the image input name and the *SegParameters.mat* file (see “Example Use Case” Section for an example). The block size, the overlap between adjacent blocks and the output folder name can also be specified if needed.

#### Morphometric Analysis

In order to analyze the results, additional tools have been developed to extract statistics in specific regions of interest (ROI), for instance the axon diameter distributions in the posterior fasciculus of the spinal cord. Other statistics are: axon and myelin areas, myelin thickness, g-ratio and axon count. These ROI can be manually drawn or can be imported from a digital version of an existing atlas (e.g., the human white matter atlas from Lévy et al., 2015). A registration module which is based on an affine 2D transformation is also provided to register the ROI to the segmentation. These operations (mask registration, labeling and metrics calculations) can be performed by a set of AxonSeg functions (see “Example Use Case” Section for more details).

### Validation

#### Data

Spinal cord images were acquired with three different imaging techniques: OM, SEM and CARS. Standard OM images were obtained from one rat and one cat (cervical sections). Samples were embedded in paraffin and imaged using a whole slide scanner with 20× magnification (Hamamatsu NanoZoomer 2.0-HT). SEM images were obtained from one rat (cervical section). Sample was stained in osmium, embedded in epoxy, polished and imaged using an SEM system (Jeol 7600F) with 1000× magnification (pixel size of 0.08 μm). CARS images were obtained from one rat (thoracic section). Sample was imaged with a 60× objective lens (UPLSAPO 1.2 NA w, Olympus) and recorded images were stitched to reconstruct the whole section (~0.2 μm/pixel). All animals were perfusion-fixed using 2% PFA and 2% Glutaraldehyde.

#### Ground Truth

Ground truth images of axon segmentations were produced by manually correcting results of AxonSeg, using the *ManualCorrectionGUI* tool. Correction included: adding missed axons, removing FPs and correcting axons shape when necessary. The resulting binary images (identifying axons as logical true and background as logical false) were used to assess segmentation quality, sensitivity and precision.

#### Sensitivity and Precision of Axon Detection

In order to evaluate the ability of AxonSeg to distinguish between true and false axon candidates, sensitivity and precision measurements were computed. TP, false negative (FN) and FP counts were obtained by automatically comparing the binary test and control images by using the centroid positions. Here, a TP is defined as a correctly identified axon (present in both test and control images), while a FN is defined as an incorrectly rejected one (present in control image but absent from the test image). We can also identify the FPs, described as axon candidates present in the test image, but absent from the control image. The sensitivity, also called TP rate (TPR), is defined as:

(1) $$TPR\;=\;\frac{TP}{TP+FN}$$

The precision, also called positive predictive value (PPV), can be defined as:

(2) $$PPV\;=\;\frac{TP}{TP+FP}$$

#### Quality of Axon Segmentation

The quality of the axon segmentations was measured by comparing segmentation results obtained from the GUI to the ground truths by using the Dice coefficient. Given two binary images *I* and *J* of the same size, we can define *a* as the number of pixels where the corresponding values of *I* and *J* are both 1 (true). In a similar way, we can also define *b* and *c* as the number of pixels where a 0 (false) value in *I* has a corresponding 1 value in *J*, and where a 1 value in *I* has a corresponding 0 value in *J*, respectively. We can then define the Dice coefficient between *I* and *J*:

(3) $$D\;=\;\frac{2a}{2a+b+c}$$

For every axon object in test image *I* and its corresponding one in the ground truth image *J* (i.e., for every TP detected), the Dice coefficient was calculated. 10th, 50th and 90th percentiles were obtained from the Dice distributions. Note that we have not quantified the quality of the myelin segmentation, as this has already been done by Begin et al. (2014).

## Results

### Sensitivity and Precision of Axon Detection

To validate the sensitivity of axon detection (TPs), three conditions were tested: axon segmentation without DA, axon segmentation with linear DA and axon segmentation with quadratic DA. The axon segmentation without DA was performed by visual assessment on a cropped region, using the feature sliders available in the GUI and trying to keep most of the TPs while eliminating as much FPs as possible. Then, linear and quadratic DAs were computed by using the same cropped region as training dataset and selecting the sensitivity/specificity value for which the Euclidean distance metric was minimal. Cropped regions of about 25% of the full image were used in all cases. Sensitivity and precision were then computed on the full images. Results are reported in Table 1 for the three available modalities (OM, SEM and CARS).

**Table 1:** **Assessment of axon detection and segmentation quality provided by AxonSeg**.

		Axon detection		Segmentation quality (from individual axon dice coefficients)		
		Sensitivity	Precision	10th Percentile	50th Percentile	90th Percentile
OM	No DA	0.8900	0.8194	0.7781	0.8636	0.9254
	Linear DA	0.8720	0.8519
	Quadratic DA	0.8857	0.8304
SEM	No DA	0.7886	0.6745	0.6876	0.8271	0.9221
	Linear DA	0.8607	0.5492
	Quadratic DA	0.8593	0.5959
CARS	No DA	0.5634	0.4751	0.7708	0.9234	0.9688
	Linear DA	0.5746	0.5126
	Quadratic DA	0.5618	0.5181

*Three modalities were tested: optical microscopy (OM), scanning electron microscopy (SEM) and coherent anti-Stokes Raman scattering (CARS). Sensitivity and precision were computed from the true positive (TP), false positive (FP) and false negative (FN) counts for three conditions: no discriminant analysis (DA) model, linear DA model or quadratic DA model. Segmentation quality was assessed by using the true positives without DA (see “Quality of Axon Segmentation” Section for Justification): 10th, 50th and 90th percentiles were computed from the Dice coefficient distribution*.

### Quality of Axon Segmentation

Quality of axon segmentation was assessed by computing the Dice coefficient between the automatic segmentation and the ground truth (segmentation with manual correction). For all modalities tested, segmentation results were produced without the use of DA, as only TPs are considered in the Dice computation and because the use of DA does not affect the quality of the segmentation (it only affects the detection of axons). Results are reported in Table 1 for the three modalities (OM, SEM and CARS).

### Application to Large-Scale Image

**Figure 4:**
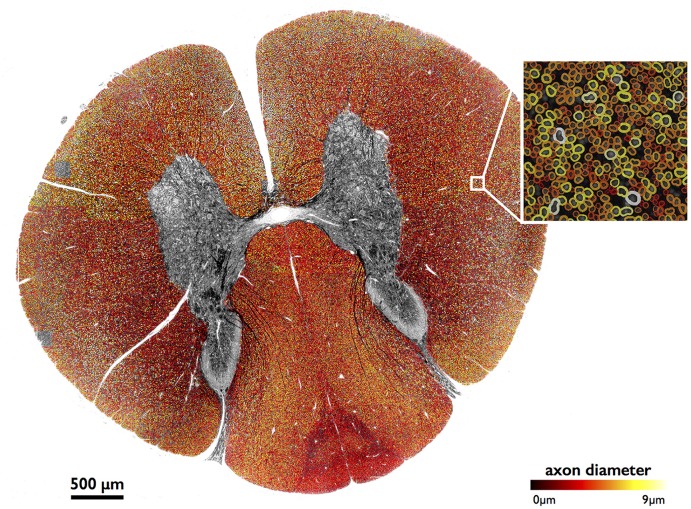
**Large-scale segmentation (axon display) in a cat spinal cord (cervical section), color-coded for axon diameter.** Myelin display (color-coded for axon diameter) is used in the zoomed region. Note that the regions outside of the white matter were masked out for better clarity, using automatic tools from AxonSeg.

Figure 4 shows results of full image segmentation (axon display) from a cervical cross-section of a cat spinal cord from OM, including a zoom in on a small region (myelin display). The displayed spinal cord slice has an area of about 4 mm^2^. Visual assessment suggests fairly good segmentation quality, with the majority of axons detected and correctly segmented. This result can also be found on our laboratory website with a zooming feature^2^. Some axons are missed, which is largely due to the poor resolution of the image.

### Atlas-Based Morphometric Analysis

**Figure 5:**
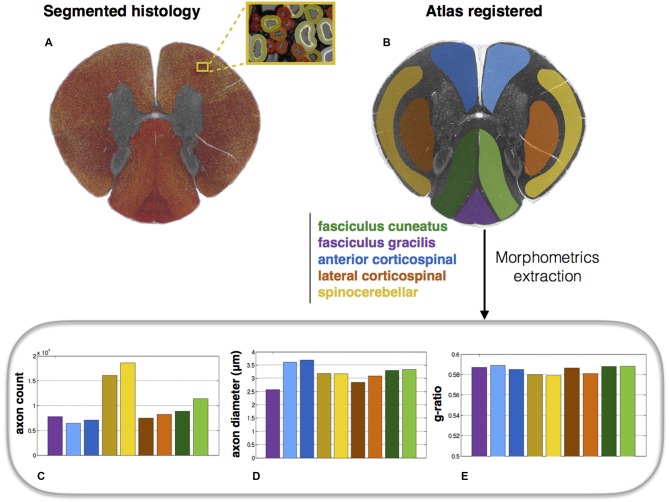
**Atlas-based morphometry from segmented images. (A)** Segmented histology results. **(B)** Atlas of white matter tracts registered to the histology image. Morphometric statistics for each of the tracts are extracted: **(C)** Axon count; **(D)** Axon diameter (mean); **(E)** Myelin g-ratio. Note the slightly lower g-ratio compared to the expected values from the literature (about 0.7; Chomiak and Hu, 2009), which is likely due to the poor resolution of the optical microscope inducing an over-segmentation of the myelin sheath.

Morphometric statistics were extracted from the same full-scale image as that in Figure 4. Results are shown in Figure 5. For each region, the following metrics were extracted: axon count, axon diameter and myelin g-ratio.

### Results in Various Imaging Modalities

**Figure 6:**
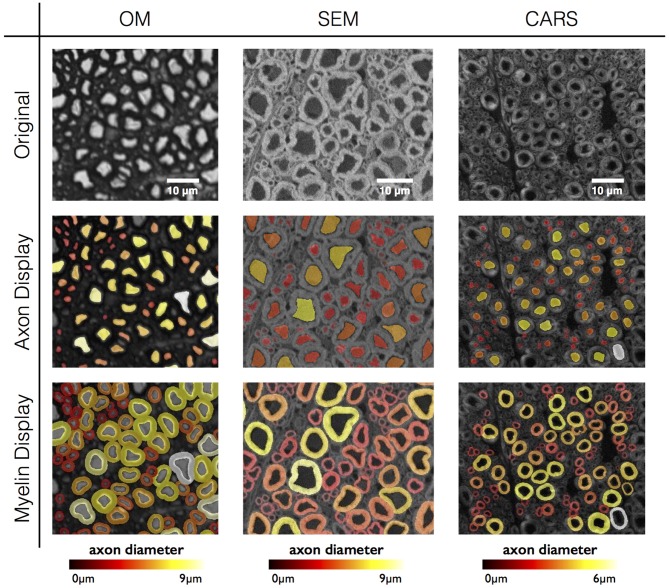
**Results in various imaging modalities: optical microscopy (OM) from a cat spinal cord, scanning electron microscopy (SEM) and coherent anti-Stokes Raman scattering (CARS) microscopy from a rat spinal cord.** All figures are color-coded for axon diameter. Note: myelin sheaths overlap more on the OM contrast due to the low resolution, inducing a blurring of the myelin sheath and therefore an apparent over-segmentation. This effect could be compensated using deconvolution algorithms applied to the image, although this will require further investigation.

As a proof of concept, the software has been tested on three different histology contrasts: OM, SEM and CARS microscopy. Figure 6 shows the results of axon and myelin segmentation, displayed using an axon diameter colormap.

### Computation Time

All data presented in this article were processed on a Macintosh (2.9 GHz Intel i5 processor, 4 cores, 8 GB 1600 MHz DDR3 RAM). The average computing time to process a single axon (including both axon and myelin segmentation) was 0.26 s. Therefore, it takes less than 8 h to automatically process a large-scale dataset (e.g., 21,000 × 12,000 pixels image, around 100,000 axons).

## Discussion

This article introduced the Axon Segmentation Toolbox: AxonSeg, a software that can segment both axon and myelin from histology images. We will now discuss the axon discrimination, validation results, computation time and results obtained from the three imaging methods assessed, and future perspectives.

### Axon Detection

AxonSeg was able to detect most of the true axons in OM and SEM images, with a sensitivity of 0.8+ in all three cases tested (see Table 1). OM presented the best detection results overall, obtaining sensitivities around 0.9 and precisions higher than 0.8 (see “Image Quality and Modalities” Section for more details). Better precision was obtained when using a DA classifier for OM and CARS images, for a similar sensitivity. The use of DA classifiers in the CARS sample increases precision by 5%, suggesting that the DA approach could be more robust than the approach without DA for this type of image. It can also be pointed out that CARS results presented lower performance overall when compared to the two other modalities. We believe this could mostly be due to the poor image quality of this particular dataset (sub-optimal fixation, sub-optimal focus, intensity inhomogeneities), the presence of a high number of very small axons (<1 μm) in the mouse spinal cord that are difficult to detect due to their size (in comparison, OM was performed on a cat cervical spinal cord, which has overall larger axons), and the similarity of intensity values between the intracellular compartment and the background, which is inherent to the CARS modality. The benefits of each modality are discussed in Section “Image Quality and Modalities”.

### Discriminant Analysis

We believe that the AxonSeg software is the first to feature a controllable DA tool allowing more flexibility on the type of contrast/modality, whereas previous software packages have set fixed parameters (e.g., axon shape and size) for detecting axons (More et al., 2011; Begin et al., 2014). Moreover, the user can decide on the ideal sensitivity/specificity depending on the application. We believe that the best way to exploit the implemented DA model is to build a template classifier by using large dataset of labeled true/false axons for each imaging modality. Therefore, the user could bypass the DA step on the cropped image and simply input one of the template classifiers available.

### Segmentation Quality

In all three modalities assessed, we observed high Dice coefficients. All Dice medians (50th percentiles) were higher than 0.8. In addition, 10th percentiles were all high, with the lower one being 0.69 (SEM), meaning 90% of axons have a Dice value higher than 0.69. After further analysis of the data, we observed that almost all the Dice coefficients lower than 0.5 come from the smaller axons, which is expected, as the small area (and thus number of pixels) used to calculate the Dice values adds bias to the measures. Note that small differences between the ground truth and segmentation results are expected, as AxonSeg produces smooth contours (as described in section “Myelin Segmentation”).

Overall, the data obtained shows only small Dice coefficient variations between different imaging techniques, demonstrating the versatility of AxonSeg. While other algorithms exist that might produce more robust segmentations (Wang et al., 2012; Liu et al., 2012), we believe that our software is a relevant addition to the existing tools, and its modularity enables other researchers to add more powerful segmentation techniques.

### Image Quality and Modalities

While AxonSeg is designed to work with a variety of modalities, further consideration is needed to properly interpret segmentation results in each of the tested modalities. OM images tend to have lower resolution than TEM (Zhao et al., 2010), which can facilitate the segmentation of axons due to the blurring of the smaller details in the intracellular compartments (e.g., mitochondria, neurotubules, etc.). However, the blurriness also induces a systematic over-segmentation of the myelin sheath, yielding a downward-bias in the g-ratio (see Figure 5). In addition, small axons (typically <1 μm) are not easily distinguishable at the OM resolution, thereby inducing an upward-bias in axon diameter distribution (Romero et al., 2000) and downward-bias in axon density. SEM images usually have higher resolution and can be noisier, therefore filtering is recommended during pre-processing. Finally, while CARS images produce exquisite specificity to myelin without the need for staining, images sometimes present some inhomogeneities within the myelin (dark patterns), especially in poorly-fixed samples (Fu et al., 2008; Schie et al., 2015). Moreover, axon and background signal have similar values, which makes it a bit more difficult to detect true axons. Filtering during pre-processing can help improving the detection.

In general, AxonSeg like any other segmentation tool strongly depends on the image contrast and resolution. Therefore, the user needs to find the optimal pre-processing parameters for their type of image before proceeding to the next steps. These parameters can then be applied to subsequent histology samples if the image contrast and resolution is kept the same.

### Computation Time and Efficiency

Computation time in AxonSeg is reasonably low, needing ~0.26 s processing time per axon. This requires about 8 h for processing 100,000 axons on standard laptop (4 CPUs, 2.9 GHz). Note that optimization is ongoing to further reduce processing time (optimal data types, sparse matrices, etc.). AxonSeg also includes a feature to automatically partition sub-sections of the full image in order to avoid RAM saturation, as explained in Section “Full Image Segmentation”.

One of the advantages of AxonSeg in terms of implementation efficiency is the *axonlist* structure: every information regarding the objects (axon and myelin segmentation, morphological statistics) is stored in a memory-efficient way, making it easier afterwards to work with the data. In that way, data on the *axonlist* can easily be processed in case the user needs to select specific fibers (e.g., based on position, diameter, and g-ratio).

### Distribution of AxonSeg

AxonSeg is an open source software, distributed under an MIT licence. It can be downloaded from either Github or MATLAB Central. It is compatible with version R2014a and does not come with compiled binaries, therefore there is no need for an OS cross-compiler. Detailed documentation on how to use AxonSeg, including example scripts, sample data and video demos, can be found on Github. Maintenance, optimization and the addition of new features are constantly made. Moreover, any researcher is welcome to contribute to the project by adding for instance other detection/segmentation methods and post-processing tools.

### Perspectives

Although AxonSeg has shown promising results, there is room for improving the quality of the axon detection and segmentation. This is a challenging task because one of the main purposes of AxonSeg is to be compatible with as many imaging modalities as possible, in order to be useful for the community at large. This article focused on presenting the main steps of AxonSeg as a proof of concept. Future work aims to explore new segmentation approaches (Markov Random Fields, Level Set), as well as other discrimination strategies, possibly integrating a machine learning module to increase robustness. A complementary approach that is being investigated is an extrapolation of segmentation metrics, robust to FNs and FPs.

Among possible applications of AxonSeg is the validation of quantitative MRI metrics from large-scale histology data, as recently demonstrated by our group (Stikov et al., 2015a,b; Duval et al., 2016).

## Example Use Case

In this section, we will present an example of workflow and some guidelines to segment an OM sample. This dataset is available when downloading the AxonSeg toolbox, under the “data” folder. Note that there is also a line-by-line tutorial available that shows more examples (*as_tutorial.m*).

### Segmentation

The AxonSeg GUI can be launched using the following Matlab command line:

SegmentationGUI test_image_OM.tif;

The pixel size for this histology sample is 0.25 μm. This value can be entered in the corresponding field. This value is important as it will be subsequently used for morphometric statistics.

In this example, axons are bright and myelin is dark. Therefore, the “Invert color” option needs to be checked. Histogram equalization is recommended in order to improve the axon-to-myelin contrast.

Due to the large size of the image (2000 × 2000 pixels), the selection of a region of interest is necessary. This can be done by drawing a small rectangle (typically 50–100 μm large) on the image using the “Crop Image” button. A preview window will zoom in this rectangle for the following steps. To go to the next step, click on “Go to next step”. To reset parameters and zoom, click on “Reset Step 0”.

In the axon segmentation step (step 1), two “ExtendedMin” sliders are available. Two different thresholds can thus be used for the extended minima algorithm if needed. Smaller threshold values do not segment correctly the whole axonal region for bigger axons, but are more efficient in order to segment the smaller ones due to the smaller contrast axon/myelin. On the other hand, higher thresholds are useful for segmentation of bigger axons, but tend to merge smaller axons together (if close to each other). Once the thresholds are set, click on “Go to next step” to merge the segmentation (logical OR) from the two sliders. Results are displayed in the preview window.

In the axon discrimination step (step 2), three sliders control different criteria (“Minimal Size”, “Solidity”, and “Ellipticity”) in order to eliminate most of the FPs. Special care is required to avoid the elimination of TP. Click on “Go to next step”.

In the post-processing and myelin segmentation step (step 3), the remaining FPs can be removed using the “Remove” button. Use left click to select axons to remove and right click to validate. Knowing these FP axons, the DA can be applied to improve the specificity of the axon detection. “Quadratic” DA usually gives better results in our tests (results not shown). Finally the “Myelin Segmentation” or “Segment full image (uncropped)” buttons perform the myelin segmentation of the cropped region and full image, respectively.

The *SegParameters.mat* structure contains all segmentation parameters after the myelin segmentation is done on the cropped image. For instance, values that can applied for the segmentation of this sample are:

PixelSize=0.25; invertColour=true;HistEq=true; Deconv=false; Smoothing=false;ExtendedMin1=47.3334; ExtendeMin2=100;minSize=6.4730; Solidity=0.8400;Ellipticity=0.2100.

Any other similar image (same resolution and contrast) can now be segmented using the following command:

as_Segmentation_full_image(’test_image_OM.tif’,’SegParameters.mat’);

### Visualization

Segmentation results can be displayed in various ways. For example, as myelin sheaths color-coded for g-ratio:

load(’axonlist.mat’);

bw_axonseg=as_display_label(axonlist,size(img),’gRatio’,’myelin’);

display=sc(sc(bw_axonseg,’hot’)+sc(img));

imshow(display);

Alternatively, both axon and myelin segmentations can be overlaid on the image, color-coded for axon diameter:

bw_axonseg_1=as_display_label(axonlist,size(img),’axonEquivDiameter’,’axon’);bw_axonseg_2=as_display_label(axonlist,size(img),’axonEquivDiameter’,’myelin’);

display=sc(sc(bw_axonseg_1,’hot’)+sc(bw_axonseg_2,’hot’)+sc(img));imshow(display);

### Statistics

Statistics can be computed using the *axonlist* structure. Below is an example to compute the axon diameter distribution:

load(’axonlist.mat’);

axon_diameters=cat(1,axonlist.axonEquivDiameter);

figure; hist(axon_diameters,50);

It is also possible to extract statistics from specific ROI using a provided RGB mask. Registration between the mask and the segmentation can be performed by running the following code:

[mask_reg_labeled, P_color]=as_reg_mask(mask,img);

Next, axon IDs belonging to each ROI of the mask are obtained with the following function:

indexes=as_stats_mask_labeled(axonlist,mask_reg_labeled);

Statistics for each ROI can be displayed as follows:

as_stats_barplot(axonlist,indexes,P_color);

## Author Contributions

AZ wrote the article. AZ and TD designed and developed the software. AG and DC contributed to the code. NS and JC-A supervised this work and wrote the article.

## Funding

This work was funded by the Canada Research Chair in Quantitative Magnetic Resonance Imaging (230815, JC-A), the Canadian Institute of Health Research [CIHR FDN-143263], the Fonds de Recherche du Québec—Santé [28826], the Fonds de Recherche du Québec—Nature et Technologies [2015-PR-182754] and the Natural Sciences and Engineering Research Council of Canada [435897-2013].

## Conflict of Interest Statement

The authors declare that the research was conducted in the absence of any commercial or financial relationships that could be construed as a potential conflict of interest. The reviewer CY and handling Editor declared their shared affiliation, and the handling Editor states that the process nevertheless met the standards of a fair and objective review.

## Footnotes

^1^Kroon (2010). Snake: Active Contour (http://www.mathworks.com/matlabcentral/fileexchange/28149-snake—active-contour), MATLAB Central File Exchange. Retrieved October 30, 2015.

^2^http://www.neuro.polymtl.ca/histology.php

## References

[B1] Alizadeh A. Dyck S. M. Karimi-Abdolrezaee S. (2015). Myelin damage and repair in pathologic CNS: challenges and prospects. Front. Mol. Neurosci. 8:35. 10.3389/fnmol.2015.00035 26283909 PMC4515562

[B2] Baker D. Amor S. (2014). Experimental autoimmune encephalomyelitis is a good model of multiple sclerosis if used wisely. Mult. Scler. Relat. Disord. 3, 555–564. 10.1016/j.msard.2014.05.002 26265267

[B3] Begin S. Dupont-Therrien O. Belanger E. Daradich A. Laffray S. De Koninck Y.. (2014). Automated method for the segmentation and morphometry of nerve fibers in large-scale CARS images of spinal cord tissue. Biomed. Opt. Express 5, 4145–4161. 10.1364/BOE.5.004145 25574428 PMC4285595

[B4] Ben-Nun A. Kaushansky N. Kawakami N. Krishnamoorthy G. Berer K. Liblau R.. (2014). From classic to spontaneous and humanized models of multiple sclerosis: impact on understanding pathogenesis and drug development. J. Autoimmun. 54, 33–50. 10.1016/j.jaut.2014.06.004 25175979

[B5] Berthold C. H. Nilsson I. Rydmark M. (1983). Axon diameter and myelin sheath thickness in nerve fibres of the ventral spinal root of the seventh lumbar nerve of the adult and developing cat. J. Anat. 136, 483–508. 6885614 PMC1171896

[B6] Chomiak T. Hu B. (2009). What is the optimal value of the g-ratio for myelinated fibers in the rat CNS? A theoretical approach. PLoS One 4:e7754. 10.1371/journal.pone.0007754 19915661 PMC2771903

[B7] Cuisenaire O. Romero E. Veraart C. Macq B. M. (1999). “Automatic segmentation and measurement of axons in microscopic images,” in Proceedings of SPIE - The International Society for Optical Engineering 3661, (San Diego, CA: Society of Photo-optical Instrumentation Engineers).

[B8] Dula A. N. Gochberg D. F. Valentine H. L. Valentine W. M. Does M. D. (2010). Multiexponential T2, magnetization transfer and quantitative histology in white matter tracts of rat spinal cord. Magn. Reson. Med. 63, 902–909. 10.1002/mrm.22267 20373391 PMC2852261

[B9] Duval T. Perraud B. Vuong M.-T. Lopez Rios N. Stikov N. Cohen-Adad J. (2016). “Validation of quantitative MRI metrics using full slice histology with automatic axon segmentation,” in Proceedings of the 24th Annual Meeting of ISMRM (Singapore).

[B10] Fu Y. Talavage T. M. Cheng J. X. (2008). New imaging techniques in the diagnosis of multiple sclerosis. Expert Opin. Med. Diagn. 2, 1055–1065. 10.1517/17530050802361161 19337386 PMC2662586

[B11] Lassmann H. (2014). Mechanisms of white matter damage in multiple sclerosis. Glia 62, 1816–1830. 10.1002/glia.22597 24470325

[B12] Lévy S. Benhamou M. Naaman C. Rainville P. Callot V. Cohen-Adad J. (2015). White matter atlas of the human spinal cord with estimation of partial volume effect. Neuroimage 119, 262–271. 10.1016/j.neuroimage.2015.06.040 26099457

[B13] Liewald D. Miller R. Logothetis N. Wagner H. J. Schüz A. (2014). Distribution of axon diameters in cortical white matter: an electron-microscopic study on three human brains and a macaque. Biol. Cybern. 108, 541–557. 10.1007/s00422-014-0626-2 25142940 PMC4228120

[B14] Liu T. Jurrus E. Seyedhosseini M. Ellisman M. Tasdizen T. (2012). Watershed merge tree classification for electron microscopy image segmentation. in Proc. IAPR Int. Conf. Pattern Recogn. 2012, 133–137. 25485310 PMC4256108

[B15] More H. L. Chen J. Gibson E. Donelan J. M. Beg M. F. (2011). A semi-automated method for identifying and measuring myelinated nerve fibers in scanning electron microscope images. J. Neurosci. Methods 201, 149–158. 10.1016/j.jneumeth.2011.07.026 21839777

[B16] Papastefanaki F. Matsas R. (2015). From demyelination to remyelination: the road toward therapies for spinal cord injury. Glia 63, 1101–1125. 10.1002/glia.22809 25731941

[B17] Romero E. Cuisenaire O. Denef J. F. Delbeke J. Macq B. Veraart C. (2000). Automatic morphometry of nerve histological sections. J. Neurosci. Methods 97, 111–122. 10.1016/s0165-0270(00)00167-9 10788665

[B18] Schie I. W. Krafft C. Popp J. (2015). Applications of coherent Raman scattering microscopies to clinical and biological studies. Analyst 140, 3897–3909. 10.1039/c5an00178a 25811305

[B19] Seidl A. H. (2014). Regulation of conduction time along axons. Neuroscience 276, 126–134. 10.1016/j.neuroscience.2013.06.047 23820043 PMC3849146

[B20] Stikov N. Campbell J. S. Stroh T. Lavelée M. Frey S. Novek J.. (2015a). *In vivo* histology of the myelin g-ratio with magnetic resonance imaging. Neuroimage 118, 397–405. 10.1016/j.neuroimage.2015.05.023 26004502

[B21] Stikov N. Campbell J. S. Stroh T. Lavelée M. Frey S. Novek J.. (2015b). Quantitative analysis of the myelin g-ratio from electron microscopy images of the macaque corpus callosum. Data Brief 4, 368–373. 10.1016/j.dib.2015.05.019 26217818 PMC4510539

[B22] Vincent L. (1998). Minimal path algorithms for the robust detection of linear features in gray images. Comput. Imaging Vis. 12, 331–338.

[B23] Wang Y. Y. Sun Y. N. Lin C. C. Ju M. S. (2012). Segmentation of nerve fibers using multi-level gradient watershed and fuzzy systems. Artif. Intell. Med. 54, 189–200. 10.1016/j.artmed.2011.11.008 22239996

[B24] Zhao X. Pan Z. Wu J. Zhou G. Zeng Y. (2010). Automatic identification and morphometry of optic nerve fibers in electron microscopy images. Comput. Med. Imaging Graph. 34, 179–184. 10.1016/j.compmedimag.2009.08.009 19796916

[B25] Zoupi L. Savvaki M. Karagogeos D. (2011). Axons and myelinating glia: an intimate contact. IUBMB Life 63, 730–735. 10.1002/iub.513 21793162

